# Assessing the factorial validity and the internal reliability of the International Trauma Questionnaire (ITQ); PTSD and complex PTSD among survivors of sexual violence in Ireland

**DOI:** 10.1017/S2045796022000245

**Published:** 2022-06-17

**Authors:** R. Frost, M. Lousion Vang, P. Hyland, M. Shevlin, A. McCarthy, J. Murphy

**Affiliations:** 1 Department of Psychology, Institute of Psychiatry, Psychology & Neuroscience, King's College London, London, UK; 2 The Collaborative Network for Training and Excellence in Psychotraumatology (CONTEXT); 3 Department of Psychology, Danish National Centre of Psychotraumatology, University of Southern Denmark, Odense, Denmark; 4 Department of Psychology, National University of Ireland, Kildare, Ireland; 5 School of Psychology, Ulster University, Derry, Northern Ireland; 6 Clinical Service Department, Dublin Rape Crisis Centre, Dublin, Republic of Ireland

**Keywords:** Post-traumatic stress disorder, sexual assault, trauma, validation study

## Abstract

**Aims:**

To assess the factorial validity and internal reliability of the International Trauma Questionnaire (ITQ) among a treatment-seeking sample of survivors of sexual violence in Ireland. In addition, to assess the diagnostic rate of post-traumatic stress disorder (PTSD) and complex post-traumatic stress disorder (CPTSD) among the samples.

**Methods:**

Participants were adult survivors of sexual violence (*N* = 114) in receipt of therapeutic support at the Dublin Rape Crisis Centre. The ITQ was utilised to measure PTSD and CPTSD symptoms and confirmatory factor analysis was employed to assess the factorial validity of the ITQ. Composite reliability was employed to assess the internal reliability of the ITQ scale scores.

**Results:**

The confirmatory factor analysis results indicated that a six-factor correlated model and a two-factor higher model were good representations of the latent structure of the ITQ, both models are consistent with the conceptualisation of CPTSD. All ITQ subscales possessed satisfactory internal reliability except for the affective dysregulation subscale. Of the sample, 56.1% met the criteria for CPTSD and 20.2% met the criteria for PTSD.

**Conclusions:**

The ITQ captured a distinction between PTSD and CPTSD symptoms and produced reliable scores within the sample, but replication with a larger sample size is required. In addition, the study findings demonstrated that CPTSD was relatively common among those seeking psychological support following sexual violence.

## Introduction

The 11th version of the *International Classification of Diseases* (ICD-11; World Health Organisation, 2018) introduced a diagnosis of complex post-traumatic stress disorder (CPTSD) in conjunction with a revised description of post-traumatic stress disorder (PTSD). ICD-11 PTSD comprises three symptom clusters including re-experiencing in the here and now, avoidance and sense of threat. CPTSD comprises these PTSD symptoms plus an additional three symptom clusters including affective dysregulation, negative self-concept and disturbances in relationships – these symptoms are collectively labelled ‘disturbances in self-organisation’ (DSO). The International Trauma Questionnaire (ITQ; Cloitre *et al*., 2018) was designed to measure these symptoms; initial findings demonstrate that the ITQ provides a valid and reliable measure of PTSD and CPTSD symptoms (Brewin *et al*., 2017; Redican *et al*., 2021). Research assessing the factorial validity of the ITQ demonstrates that the latent structure is best represented by two models: (i) a correlated six-factor model where the factors reflect the six PTSD and DSO symptom clusters, and (ii) a two-factor higher-order model where the first-order factor correlations are explained by two second-order factors reflecting PTSD and DSO (Brewin *et al*., 2017; Redican *et al*., 2021). Sexual trauma is associated with an increased risk of CPTSD (Cloitre *et al*., 2013; Hyland *et al*., 2017*a*) but few studies have assessed the factorial validity of the ITQ among survivors of sexual violence in receipt of therapeutic support. It is necessary to conduct such research to ensure that the ITQ provides a valid measure of PTSD and CPTSD symptoms in this context, especially if this is a context where the symptoms of both disorders are likely to emerge.

Sexual violence is relatively common in Irish society (McGee *et al*., 2002). Among a nationally representative sample of Irish adults, it has been estimated that 15% of people have experienced rape at some point in their life and 31% have been exposed to some form of sexual violence (e.g. sexual harassment; Vallières *et al*., 2020). Moreover, rates of ICD-11 PTSD (8.0 *v.* 3.6%) and CPTSD (14.2 *v.* 4.9%) are significantly higher among those who have experienced sexual violence. At present, there is no data concerning the rate of ICD-11 CPTSD and PTSD among survivors of sexual violence who have sought psychological support in Ireland. International figures suggest that approximately 60% of treatment-seeking individuals meet the criteria for a diagnosis of either ICD-11 PTSD or CPTSD, with most meeting the criteria for CPTSD (Hyland *et al*., 2017*b*; Vang *et al*., 2021). Up-to-date information regarding the psychological impact of sexual violence in Ireland (and other jurisdictions) is required to acknowledge the mental health needs of victim survivors.

The present study aimed to (i) assess the factorial validity and the internal reliability of the ITQ, and (ii) assess the diagnostic rate of ICD-11 PTSD and CPTSD among an adult treatment-seeking sample of survivors of sexual violence in Ireland. Based on the research literature, it was hypothesised that the ITQ would provide a valid measure of PTSD and CPTSD symptoms – both the six-factor correlated model and the two-factor higher-order model would provide a satisfactory representation of the latent structure of the ITQ in this sample (Brewin *et al*., 2017; Redican *et al*., 2021). It was also hypothesised that a higher proportion of individuals would meet the criteria for CPTSD as compared to PTSD (Hyland *et al*., 2017*b*; Vang *et al*., 2021).

## Method

### Participants and procedure

The study was of a cross-sectional quantitative design. Participants (*N* = 114) comprised of a convenience sample of individuals in receipt of therapeutic services at the Dublin Rape Crisis Centre (DRCC). The DRCC provides counselling services to those 16 years and older who have experienced sexual assault in childhood or adulthood. Due to ethical considerations only adult service-users (⩾18 years) were eligible to participate in this study. Data was collected from participants who had spent different lengths of time receiving therapy – this ranged from 0 to 180 months (mode = 12 months). Prior to participation, service users were provided with an information sheet outlining (i) the purpose of the data collection; (ii) the topics measured; (iii) the voluntary nature of participation and (iv) the confidentiality of response data. There were no incentives or rewards for participation. Data was collected from March 2019 to February 2020. Data was gathered via a paper and pencil self-report questionnaire. Individuals provided information about demographic characteristics including age, sex, nationality, relationship status, level of education and employment status. The experience of sexual assault across the lifespan was approximated using an item from the Life Events Checklist (Weathers *et al*., 2013); ‘Sexual assault (rape, attempted rape, made to perform any type of sexual act through force or threat of harm)’. Participants were asked two questions in relation to this item: (i) whether they had experienced sexual assault in childhood (<18 years) and (ii) whether they had experienced sexual assault in adulthood (⩾18 years). Each item was scored in a binary response format: ‘Yes/presence = 1’ or ‘No/absence = 0’.

### ICD-11 PTSD and CPTSD

The ITQ (Cloitre *et al*., 2018) contains six items to measure the three PTSD symptom clusters and six items to measure the three DSO symptoms clusters – each symptom cluster is measured by two items. Six items measure functional impairment, three items in relation to PTSD and DSO symptoms, respectively. The functional impairment items assess symptom impact on (i) relationships and social life, (ii) work or ability to work and (iii) other important aspects of life such as parenting, school or college work. All items are based on a five-point Likert scale ranging from 0 (‘not at all’) to 4 (‘extremely’), and a score ⩾2 (‘moderately’) indicates symptom endorsement. Diagnosis of PTSD requires that one symptom is present from each PTSD cluster plus the endorsement of at least one functional impairment item. Diagnosis of CPTSD requires that PTSD criteria are satisfied, plus a symptom from each of the DSO symptom clusters is present and accompanied by the endorsement of an item of functional impairment. The ICD-11 taxonomic structure only permits a diagnosis of PTSD or CPTSD; if an individual meets the criterion for CPTSD that person does not qualify for a PTSD diagnosis.

### Data analysis

Confirmatory factor analysis (CFA) was utilised to test the factorial validity of the ITQ. The correlated six-factor and higher-order models described in the introduction were tested. These models were estimated using the robust maximum likelihood estimator in Mplus 7.0 (Muthén and Muthén, 2012). Acceptable model fit was indicated by a non-significant chi-squared (*χ*^2^) value, values ⩾0.90 for the comparative fit index (CFI) and Tucker–Lewis index (TLI; Tucker and Lewis, 1973; Bentler, 1990) and a root mean square error of approximation (RMSEA; Steiger, 1990) values ⩽0.06. Composite reliability was used to estimate the internal reliability of the scale scores, and this is preferable to Cronbach's alpha because it does not assume tau-equivalence (Raykov, 1997). At least ten cases per indicator variable or *N* = 150 have been recommended as a reasonable sample size for CFA (Nunnally, 1978; Muthén and Muthén, 2002). The relatively small sample size in the present study has been considered in the interpretation of the study findings.

## Results

The sample (*N* = 114) was mostly Irish (*n* = 96, 84.2%), primarily identified as female (*n* = 101, 88.6%), with an age range of 18–74 years (*M* = 35.7, s.d. = 13.3). Most participants were not in a committed relationship (*n* = 80, 70.2%), 50.9% reported being in full-time or part-time employment (*n* = 56), and 38.6% had a bachelor's degree or a higher level of academic qualification (*n* = 44). Of those who disclosed the nature of sexual assault (*n* = 87), 55.6% experienced sexual violence in childhood and 81% experienced sexual violence in adulthood; 31% experienced sexual violence in both childhood and adulthood.

The frequency of item endorsement (see Table 1) was high, even the least frequently endorsed item, a DSO item measuring negative self-concept ‘I feel worthless’, was endorsed at 66.7%. The most frequently endorsed item was a PTSD item measuring a heightened sense of threat ‘Being “super-alert”, watchful, or on guard?’ (92.1%).

**Table 1.** Frequency of CPTSD item endorsement

Item	Total (%)
Post-traumatic stress disorder	
Having upsetting dreams that replay part of the experience or are clearly related to the experience? (Re1)	67.5
Having powerful images or memories that sometimes come into your mind in which you feel the experience is happening again in the here and now? (Re2)	76.3
Avoiding internal reminders of the experience (for example, thoughts, feelings, or physical sensations)? (Av1)	86.8
Avoiding external reminders of the experience (for example, people, places, conversations, objects, activities, or situations)? (Av2)	89.5
Being ‘super-alert’, watchful, or on guard? (Th1)	92.1
Feeling jumpy or easily startled? (Th2)	79.8
Disturbance in self-organisation	
When I am upset, it takes me a long time to calm down (Ad1)	82.5
I feel numb or emotionally shut down (Ad2)	76.3
I feel like a failure (Nsc1)	78.1
I feel worthless (Nsc2)	66.7
I feel distant or cut off from people (Dr1)	79.8
I find it hard to stay emotionally close to people (Dr2)	73.7

%, percentage of item endorsement; item endorsement ⩾2; Re, Re-experiencing; Av, Avoidance; Th, Sense of Threat; Ad, Affective Dysregulation; Dr, Disturbed Relationships; Nsc, Negative Self-Concept.

**Fig. 1.**
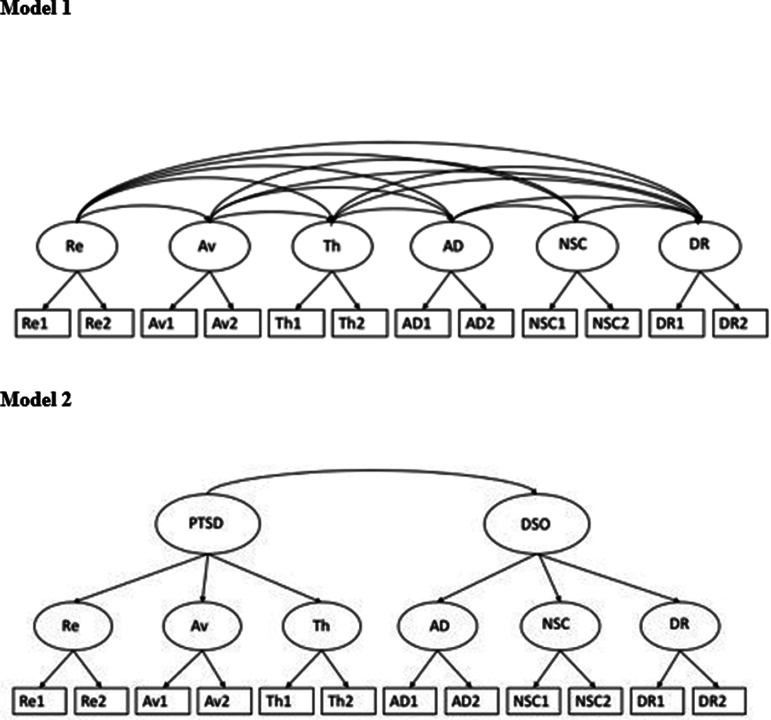
CFA models testing the latent structure of the ITQ.

The correlated six-factor model (*χ*^2^(39) = 43.94, *p* < 0.001; RMSEA (95% CI) = 0.033 (0.00–0.08); CFI = 0.991; TLI = 0.984; AIC = 3843; BIC = 3983) and the higher-order model (*χ*^2^(47) = 48.63, *p* < 0.001; RMSEA (95% CI) = 0.017 (0.00–0.07); CFI = 0.997; TLI = 0.996; AIC = 3834; BIC = 3952) provided a good fit to the data. Both models provided a close approximation of the sample data and are both viable models of the latent structure of the ITQ (see Fig. 1). In both model 1 (see Table 1) and model 2 (see Table 2), all items loaded significantly onto their respective first-order factors, in both models one item ‘Dr 1’ had a loading above 1.0. In model 1, significant correlations were found between all first-order factors ranging from *r* = 0.26 to *r* = 0.71 (see Table 2). In model 2, all first-order factors loaded significantly onto the second-order factors (see Table 3) and the correlation between the PTSD and DSO factors was high (*r* = 0.75).

**Table 2.** Standardised factor loadings and factor correlations for model 1

	Re	Av	Th	Ad	Nsc	Dr
Items						
Re1	0.73					
Re2	0.79					
Av1		0.91				
Av2		0.58				
Th1			0.79			
Th2			0.90			
Ad1				0.40		
Ad2				0.82		
Nsc1					0.86	
Nsc2					0.94	
Dr1						1.13
Dr2						0.61
Factors						
Av	0.71					
Th	0.66	0.48				
Ad	0.70	0.65	0.47			
Nsc	0.58	0.48	0.33	0.74		
Dr	0.43	0.40	0.26	0.58	0.59	

Re, Re-experiencing; Av, Avoidance; Th, Sense of Threat; Ad, Affective Dysregulation; Dr, Disturbed Relationships; Nsc, Negative Self-Concept.

All loadings were statistically significant (*p* < 0.05).

**Table 3.** Standardised factor loadings for model 2

	Re	Av	Th	Ad	Nsc	Dr
Items						
Re1	0.72					
Re2	0.80					
Av1		0.89				
Av2		0.59				
Th1			0.81			
Th2			0.88			
Ad1				0.39		
Ad2				0.82		
Nsc1					0.86	
Nsc2					0.94	
Dr1						1.11
Dr2						0.63
Factors	PTSD	DSO				
Re	0.95					
Av	0.78					
Th	0.66					
Ad		0.90				
Nsc		0.84				
Dr		0.68				

Re, Re-experiencing; Av, Avoidance; Th, Sense of Threat; Ad, Affective Dysregulation; Dr, Disturbed Relationships; Nsc, Negative Self-Concept.

All loadings were statistically significant (*p* < 0.05).

The composite reliability estimates indicated acceptable levels of internal reliability for all subscales apart from the affective dysregulation subscale: Re-experiencing = 0.73, Avoidance = 0.84, Sense of Threat = 0.73, Affective Dysregulation = 0.56, Negative Self-Concept = 0.90 and Disturbed Relationships = 0.90. The PTSD (0.81) and DSO (0.84) subscales also had acceptable levels of internal reliability.

Overall, 76.3% of the sample met the diagnostic criteria for either ICD-11 PTSD or CPTSD; 20.2% met the criteria for PTSD and 56.1% met the criteria for CPTSD.

## Discussion

The two-factor higher-order model and the correlated six-factor model both provided a good representation of the latent structure of the ITQ. Both models capture the distinction between PTSD and DSO symptoms inherent to the ICD-11 formulation of PTSD and CPTSD (WHO, 2018) among the sample of survivors of sexual violence. These findings add to the research literature indicating that the ITQ may be used to measure PTSD and DSO symptoms at a cluster-specific level or a broader domain level (Brewin *et al*., 2017; Redican *et al*., 2021). The ITQ scale scores possessed acceptable levels of internal reliability, except for affective dysregulation. The two items utilised to measure affective regulation may reflect opposing types of emotion regulation strategies (hypo-affective and hyper-affective responses), individuals may primarily rely on one of these strategies (Sele *et al*., 2020), which may account for a lower level of internal reliability. In addition, an item loading greater than 1 was observed, while it is possible that this is related to the relatively small sample size (Kyriakos, 2018) it is not unusual to observe factor loadings greater than 1.0 (Deegan, 1978). This does not necessarily indicate model misspecification but rather may indicate highly correlated factors (Deegan, 1978). The CFA findings are consistent with the findings of previous studies (Brewin *et al*., 2017; Redican *et al*., 2021), but further replication is warranted among this population with a larger sample size.

Approximately three-quarters of the sample met the diagnostic criteria for either ICD-11 PTSD or CPTSD. Participants were assessed at various stages in their therapeutic journey, had participants been assessed at the point of initial assessment estimates concerning the diagnostic rate of PTSD and CPTSD may have been higher. These results are similar to observations in other clinical samples (Hyland *et al*., 2017*b*; Vang *et al*., 2021). A higher proportion of the sample met the criteria for CPTSD as compared to PTSD (56.1 *v.* 20.2%), this finding is also in keeping with research demonstrating that CPTSD is more likely to occur in the context of sexual violence (Cloitre *et al*., 2013; Hyland *et al*., 2017*a*). However, further research is warranted to evaluate the association between various types of trauma exposure and CPTSD among samples that have experienced sexual violence. Clinicians working with individuals who have experienced sexual violence are more likely to encounter service users with CPTSD. Research demonstrates that compared to PTSD, CPTSD is associated with higher levels of functional impairment and may require additional clinical support (Karatzias and Cloitre, 2019).

Overall, the findings provide support for the factorial validity and internal reliability of the ITQ as a measure of PTSD and CPTSD in this trauma context. Regular and up-to-date estimates concerning the prevalence and impact of sexual violence are required to highlight the psychological impact of this public health problem.

## Availability of data and materials

Due to the nature of this research, participants of this study did not agree for their data to be shared publicly, so supporting data are not available.
